# Personal

**Published:** 1890-08

**Authors:** 


### Personal.

Dr. William J. Conklin, of Dayton, was elected president of the Ohio State Medical Society at its annual session in Columbus in June. Dr. Conklin departed for Europe July 17th ultimo, to attend the Tenth International Medical Congress in Berlin. He will return early in September.

---

Dr. Elmer E. Starr, of Buffalo, sailed for Europe in July. It is announced that he will remain abroad for about six months, and spend a portion of the time wheeling through France and Germany.

DR. GEORGE T. WETMORE, of New York, has recently paid a visit to Buffalo in the interest of the New York Physicians' Mutual Aid Association. During the few days Dr. Wetmore remained in the city, we understand he increased the membership of this worthy Association with upwards of fifty names. Considering the fact that this is the vacation season, when so many physicians are either out of the city, or are declining any business except such as strictly pertains to their profession, we regard this as a very creditable showing; and we hope that every one of the Buffalo members will consider it a pleasure and a *duty* to retain affiliation with the Physicians' Mutual Aid Association during the remainder of their lives.

---

MRS. LYMAN ABBOTT, wife of the successor to Henry Ward Beecher as pastor of Plymouth Church, is to become one of the editors of *The Ladies' Home Journal*, on September 1st, next.

